# Cytokines and immunologic checkpoint molecules in predicting success of allergen immunotherapy

**DOI:** 10.1038/s41598-026-53894-6

**Published:** 2026-05-18

**Authors:** Martin Berge, Olof Hultgren, Svante Hugosson, Robert Kruse, Amanj Saber

**Affiliations:** 1 Department of Otolaryngology, School of Medical Sciences, Faculty of Medicine and Health, Örebro University, Örebro, Sweden; 2 Department Clinical Immunology and Transfusion Medicine, School of Medical Sciences, Faculty of Medicine and Health, Örebro University, Örebro, Sweden; 3 Department of Clinical Research Laboratory, School of Medical Sciences, Faculty of Medicine and Health, Örebro University, Örebro, Sweden

**Keywords:** Allergen immunotherapy, Cytokines, Immunological checkpoint molecules, Machine learning, Clustering, Biomarkers, Computational biology and bioinformatics, Immunology, Medical research

## Abstract

There are large variations in how individual patients respond to allergen immunotherapy (AIT) against grass and/or birch allergy. There are currently no reliable biomarkers to predict which patients are likely to benefit from the treatment. The purpose of this study was to examine the potential of cytokine and soluble immunologic checkpoint molecule (ICM) levels as biomarkers for AIT success. Blood samples collected before starting AIT were analyzed for concentration of 92 different cytokines and 14 different ICMs. Traditional univariable statistical analysis was performed to evaluate differences between responders and non-responders. Furthermore, both unsupervised and supervised machine learning algorithms were used for multivariable analysis of differences between the responders and non-responders and to try to identify clusters within the subjects which could potentially be linked to endotypes of allergic rhinitis. Neither univariable nor multivariable analysis showed any significant correlations between AIT outcome and pre-treatment levels of cytokines or soluble ICM. In the cluster analysis, 4 clustering algorithms consistently grouped 48 of the 60 subjects into 3 distinct clusters. However, these clusters did not correlate with clinical characteristics, indicating that the clusters are unlikely to represent actual biological endotypes. The findings of this study did not provide evidence supporting the use of pre-treatment levels of cytokines and ICM as biomarkers for AIT outcome.

**Supplementary Information:**

The online version contains supplementary material available at 10.1038/s41598-026-53894-6.

## Introduction

Allergic rhinitis (AR) is a mainly Th2-driven inflammatory disease, manifesting as a chronic upper airway disease. As it affects up to 30% of the population it poses a considerable socio-economic burden^1^. There are plenty of symptomatic treatments for AR, ranging from over-the-counter medications, such as oral antihistamines and intranasal corticosteroids, to prescription drugs such as leukotriene receptor antagonists. However, the only potentially disease-modifying treatment of AR is allergen immunotherapy (AIT)^2^.

The current recommendations for the treatment of AR follow a step-up regime of different pharmacological medications, depending on the severity of the patient’s symptoms. If the patient is still reporting severe symptoms despite adequate pharmacological treatment, they should be considered for AIT^3^. However, not all patients respond equally to AIT, and some patients may experience only a small or even a complete lack of effect from the treatment^4–6^. Although new methods for administering AIT, such as sublingual AIT, have somewhat relieved the need for repeated health care visits compared to the classical subcutaneous AIT, it still requires a lengthy treatment for several years before the effect can be completely evaluated. Better methods for selecting the patients likely to benefit from AIT would result in benefits both for the individual patient as well as for the healthcare providers, by limiting the number of patients who undergo a lengthy and costly treatment with a small chance for improvement.

Furthermore, AIT has shown potential in preventing the disease progression of AR if applied at an early stage^7^. Therefore, the identification of biomarkers to successfully select patients who would benefit from AIT might enable offering patients AIT at an earlier stage, and in turn prevent patients with AR from progressing into a more severe form of the disease, such as bronchial asthma. An earlier study by the authors has examined allergen specific immunoglobulin E (IgE) as a potential biomarker for predicting AIT-response. However, no such connection could be found^6^.

With the current understanding of the pathophysiology of AR, cytokines are known to modulate the immune response to an allergen. For example, type 2 cytokines such as IL-4 and IL-13, are known to be of importance in the pathophysiology of allergic inflammation^8^. Immunologic checkpoint molecules (ICM) are another group of molecules that plays a role in regulating the response of immunologic T-cells. In allergic inflammation, programmed cell death protein 1 (PD-1) and its ligands have been proposed to play a role in regulating the immune response^9,10^.

In addition to their roles in allergic inflammation, cytokines have been proposed as a potential biomarker to monitor the effect of AIT. While some studies have demonstrated a decrease in Th2-cytokines (for example IL-4, IL-13, and IL-9) and an increase in Th1-cytokines (such as IFN-γ and IL-12) during AIT, the results have been inconsistent and there is yet to be shown a correlation between change in cytokine levels and AIT-result^11^. Furthermore, novel treatments directly targeting cytokine signaling pathways have shown potential in the treatment of other inflammatory diseases, while their effect on AR is still unclear^12,13^.

The roles of ICM in AIT are much less studied compared to cytokines. However, one study has shown that the cellular expression of the regulatory ICM CTLA-4 was increased during AIT against house dust mites^14^. They could also show an increase in the expressions of ICMs LAG-3 and PD-1, although these were not statistically significant. While these results do not directly support the use of ICM in a pre-treatment setting to predict AIT success, they suggest that ICMs may be involved in immune regulation during AIT. Furthermore, the same study also found a strong correlation between the cellular expressions and the levels of soluble ICM in serum.

Traditionally, the selection of which treatment should be offered which patient is based on the patient’s phenotype, which is based on observable characteristics such as symptoms. However, it is likely that to better understand the differences in efficacy of different anti-allergic treatments the classification of patients into phenotypes is insufficient. Therefore, categorizing patients based on the underlying pathophysiological mechanisms, so-called endotypes, may facilitate a precision-based approach in caring for these patients^15^. For example, an earlier study has presented the cytokines interleukin-10 (IL-10) and IL-33 as potential biomarkers for efficacy of sublingual AIT against house dust mites^16^.

The aim of this study is to examine cytokines and ICM as potential pre-treatment predictors for AIT-success in patients with grass and/or birch allergy. A secondary aim is to see if we can find clusters within the subjects, that can be linked to potential endotypes of AR.

## Materials and methods

### Study design and population

This is a retrospective study based on a cohort of 128 adult patients who were set to undergo AIT targeting grass and/or birch allergy. The inclusion of subjects has been described in a previous publication^6^. These data contain information about clinical improvement after AIT (based on patient-reported symptoms before and after AIT), demographic characteristics, results from skin prick testing (SPT), and concentrations of allergen specific IgE.

Due to both practical and economic limitations of the current study, not all 128 subjects could be analyzed for cytokine and ICM-levels. Therefore, 30 subjects who responded to AIT were matched with 30 non-responders using the R-package MatchIt^17^. We used exact matches for sex and optimal matches for age. Serum samples were taken from the subjects before they started AIT. The samples were stored in a bio bank at − 20 °C, until they were then further analyzed for cytokines and soluble ICM.

All patients have given informed consent to be included in the study. This study has received approval by the Swedish Ethical Review Authority. The study and all methods included have been performed according to the relevant guidelines and regulations.

### Cytokine analysis

Serum samples were analyzed using Proximity Extension Assay technology, provided by Olink (Olink Proteomics, Uppsala, Sweden). The samples were analyzed with the Olink® Target 96 panel, which provides analysis of 92 protein biomarkers (Supplement 1). The Olink Proseek Multiplex antibody-based methodology proximity extension assay (PEA) has been described in detail elsewhere^18^. Limit of detection (LOD) and specificity can be obtained via Olink’s website (http://www.olink.com). Data was obtained and presented as relative protein levels, Normalized Protein eXpression (NPX) values, on log2 scale.

### Immunologic checkpoint molecules analysis

Samples were analysed with a multiplex assay, using Luminex xMAP technology (ProcartaPlex™ Human Immuno-Oncology Checkpoint Panel 1, 14plex, *ThermoFisher*, USA). Analysis was conducted in accordance with the manufacturer’s instructions. Serum concentration was determined for 14 different immunologic checkpoint molecules (Supplement 2). Data was obtained and presented as protein concentration in pg/ml.

### Statistics

Microsoft® Excel (Microsoft, Seattle, W.A., USA) was used to store the data. Tables were created using R, a software environment for statistical computing^19^. Age is presented as median (IQR), and categorical variables as frequencies (percentage).

Prior to univariable analysis, normality was tested using the Shapiro–Wilk test. As approximately half of the proteins deviated from a normal distribution, we systematically employed the non-parametric Wilcoxon rank-sum test and *P* values were adjusted for multiple comparisons using a false discovery rate (FDR) approach with q-values reported. Proteins were regarded as being significantly differentially regulated if they showed a fold change > 1.2 and a q-value of < 0.05.

Unsupervised multivariable dimensionality reduction of protein levels was performed in R with principal component analysis (PCA) to evaluate variation in protein levels with regards to AIT-outcome, sex, asthma diagnosis, target of AIT, and pre-AIT treatment.

Supervised multivariable machine learning was performed in R to investigate if AIT-outcomes could be separated with regards to their protein levels and/or immunologic checkpoint molecules. The machine learning was performed with a nested cross-validation workflow assessing proteins as predictors and classifiers of study group with five different machine learning algorithms.

Within each randomized nest the study cohort was partitioned into training (75%) and testing (25%) sets. The concentration of cytokines, concentration of cytokines and ICM combined with clinical characteristics (for the sub-cohort of 60 subjects), or concentration of allergen-specific IgE combined with clinical characteristics (for the full cohort of 128 subjects) of the training set was either used as a full set of all variables or reduced by Boruta selection with a cut-off of 0.01 (colino package in R).

Each set of data was standardized, and bagged tree model imputation of missing data (recipe package in R) and class balancing with Adaptive Synthetic Algorithm minority oversampling technique (themis package in R) was performed on the training set. Thereafter, penalized regularized logistic regressions (glmnet package in R), Random forests (ranger package in R), Neural network (nnet package in R), Naïve Bayes (klaR package in R) and lightGBM (bonsai package in R), were in parallel trained with hyperparameter tuning with a Latin Hypercube search approach with internal validation on 20 bootstraps of the training data.

Final model performance was assessed on the testing data. To estimate the robustness of predictions with regards to random effects of partitioning, this workflow procedure was repeated iteratively 20 times as randomized nested cross-validation with random partitioning of samples to training and testing set at each iteration. The variability of model performances from the nested cross-validations was estimated by fitting Bayesian models and Markov Chain Monte Carlo via the tidyposterior and rstanarm packages in R, with 5000 iterations, four chains and a prior normal distribution for the random (nest) intercepts. Posterior probability distributions of mean area under curve (AUC) and their contrasted differences between all models were evaluated for region of practical equivalence.

Finally, four different clustering algorithms (CLARA, Hierarchical Clustering, Fuzzy C-means clustering, and Mclust) were used to create clusters of subjects based on their protein expressions. The results from the different algorithms were then compared to create consensus clusters, which were then analyzed to see which proteins differed between the clusters.

## Results

### Baseline characteristics

For 6 subjects in the original study cohort, there were insufficient serum volumes remaining for further analysis. From the remaining 122 subjects, 30 subjects who had responded to AIT were randomly selected. They were then matched, based on age and sex, with 30 subjects who had not responded to AIT. The baseline characteristics of the sub-cohort selected for further cytokine and ICM-analysis are presented in Table 1. There were no clear differences in the clinical characteristics between the improved and non-improved groups.

**Table 1:** Baseline characteristics of the sub-cohort. Categorical variables are presented as frequency (percentage), and age as median (IQR).

Characteristic	Improved	
	No (n = 30)	Yes (n = 30)
Sex		
Female	19 (63%)	19 (63%)
Male	11 (37%)	11 (37%)
Age	28 (20–38)	28 (21–38)
Target allergen		
Birch	6 (20%)	5 (17%)
Grass	8 (27%)	10 (33%)
Grass + Birch	16 (53%)	15 (50%)
Asthma	8 (27%)	8 (27%)
Self-reported asthma	15 (50%)	13 (43%)
Pre-AIT treatment		
Oral antihistamines	27 (90%)	25 (83%)
Intranasal corticosteroids	25 (83%)	20 (67%)
Systemic corticosteroids	9 (30%)	10 (33%)
Skin prick test		
Birch	4 (13%)	2 (7%)
Grass	6 (20%)	3 (10%)
Grass + Birch	19 (63%)	25 (83%)
Unknown	1 (3%)	0 (0%)

### Analysis of immune proteins

**Fig. 1:**
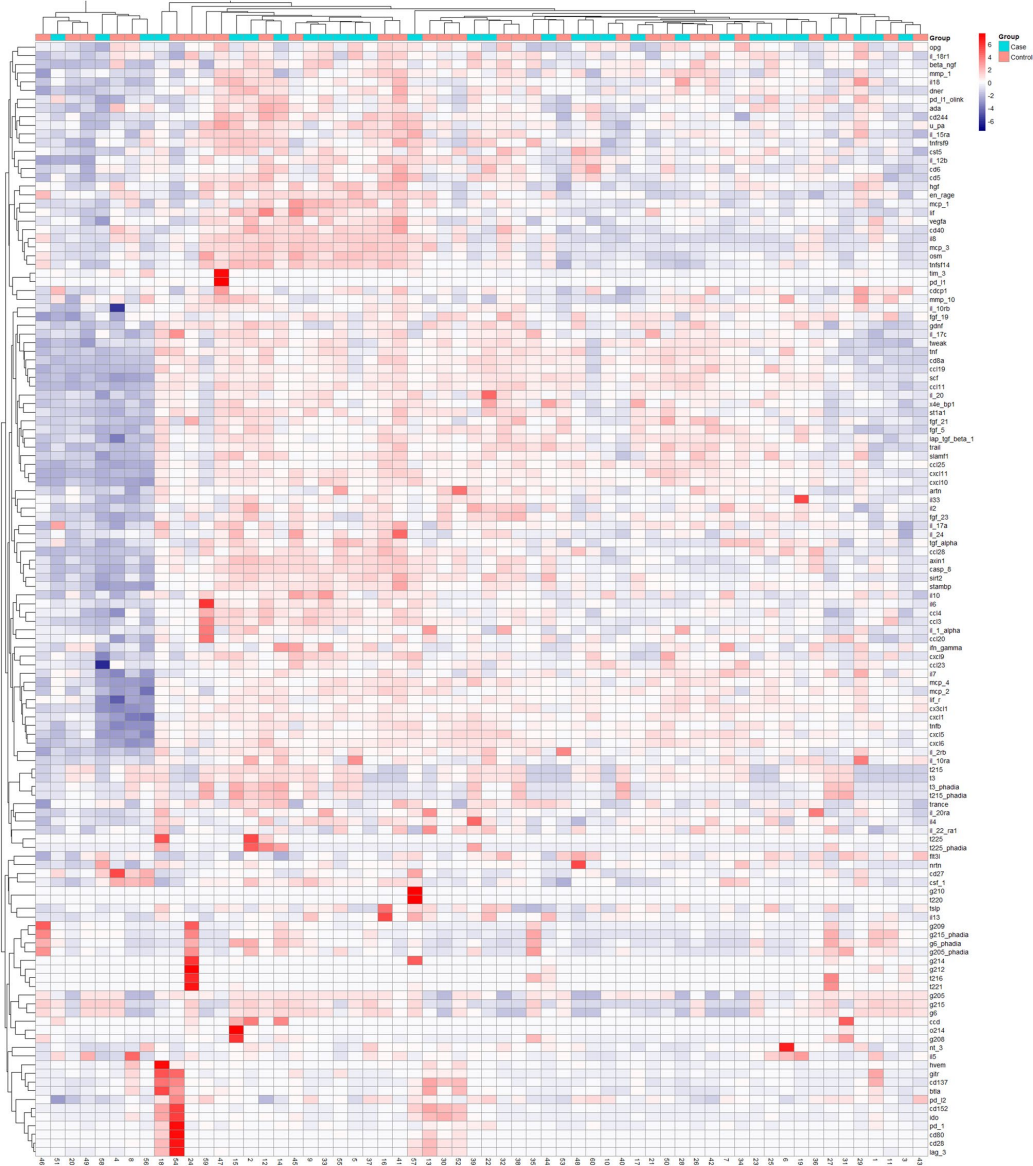
Hierarchical clustered heat map of Olink, ICM and sIgE protein abundance. Individual values are centered and scaled using z-score standardization and clustered by AIT-outcome (columns; improved—teal and non-improved—pink) and proteins (rows). Gradients in the heatmap corresponds to protein concentration (blue: low, red: high). No distinct clusters of improved versus non-improved subjects are seen. *ICM* immunologic checkpoint molecule, *sIgE* allergen specific IgE, *AIT* allergen immunotherapy.

Sera from the sub-cohort were then analyzed for 92 different proteins using Olink® Target 96 panel, and 14 ICM proteins using ProcartaPlex™ Human Immuno-Oncology Checkpoint Panel 1. A hierarchical clustered heatmap was used to visualize any potential association between protein concentrations and AIT-outcome. No distinct clusters were found associated with AIT-outcomes (Fig. 1). The analysis of ICM-concentrations showed great variation between subjects, but also between different ICMs (Supplement 3).

**Fig. 2:**
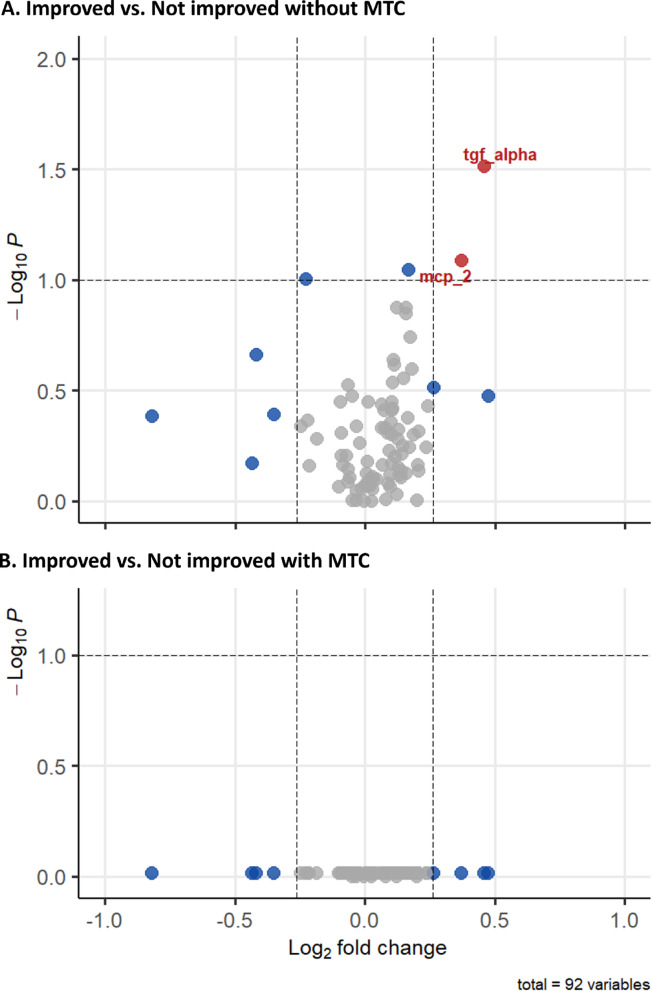
Volcano plot difference in Olink-protein and ICM concentration between improved and non-improved subjects. Proteins considered as different with fold change > 1.2 and alpha set to < 0.1. (**A**) Before correction for multiple testing. (**B**) After multiple testing correction with Benjamini–Hochberg. Colors are representing significance (red: *P* < 0.1 and fold change > 1.2; blue: *P* < 0.1 or fold change > 1.2). After correction for multiple comparisons, no proteins are significantly different.

When conducting univariable analysis of Olink-proteins and ICM relative to AIT-outcome, no statistically significant differences could be seen after correcting for multiple testing (Fig. 2). Before multiple testing correction, transforming growth factor alpha (TGF-alpha) and monocyte chemoattractant protein 2 (MCP-2) were shown to be elevated in the improved versus non-improved group. However, when directly comparing the groups, there is significant overlap in protein levels (Supplement 4). Likewise, no differences could be seen in the levels of IL10 and IL33 between responders and non-responders. Furthermore, no statistically significant differences were found between improved and non-improved subjects upon univariable analysis of ICM.

### Machine learning analysis

**Fig. 3:**
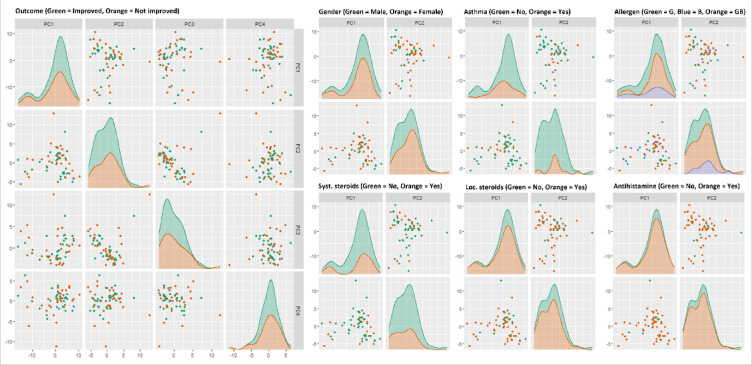
PCA-plot of differences in Olink-protein concentration relative to AIT-outcome and baseline characteristics. Unsupervised distribution of variation in protein abundance in samples overlaid with color representing AIT-outcome, sex, asthma diagnosis, target of AIT, and pre-AIT treatment. No noticeable effects on variation.

Unsupervised, multivariable analysis with PCA showed no separation in PC1-4 between improved and non-improved subjects based on concentration of Olink-proteins (Fig. 3). Furthermore, PC1-2 did not separate the subjects based on sex, asthma diagnosis, target of AIT, or pre-AIT treatment.

**Fig. 4:**
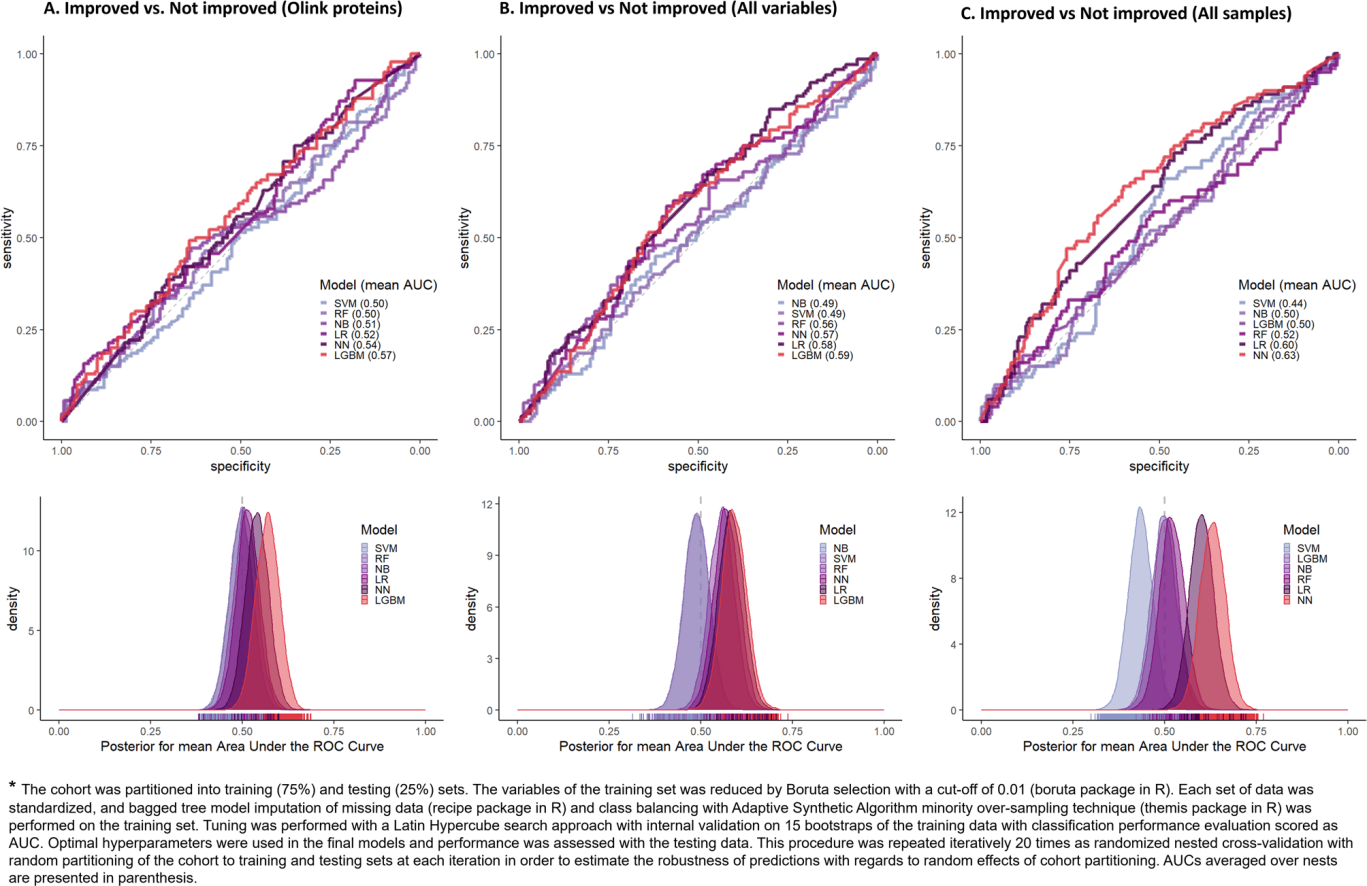
Machine learning classification of improved versus non-improved subjects. Upper sections show the averaged ROC curve (mean area under the curve (AUC) within parenthesis), and lower sections show the distributions of posteriors for mean AUC from the nests in nested cross-validation for the respective algorithms. (**A**) Performance with Olink proteins in sub-cohort (60 samples). (**B**) Performance with all variables (Olink-proteins, ICM, clinical characteristics and allergen-specific IgE variables) in sub-cohort (60 samples). (**C**) Performance with clinical characteristics and allergen specific IgE variables in full cohort (128 samples). Colors represent different machine learning algorithms. *LR* penalized regularized logistic regressions, *RF* random forests, *NN* neural network, *NB* naïve bayes, *LGBM* lightGBM models.

To evaluate multivariable patterns associated with AIT-outcome different machine learning algorithms for classification were trained using optimal hyperparameters on bootstrap-data in random nested cross-validation performed 20 times. The models were run using AIT-success as the outcome variable relative to either concentration of cytokines alone, or concentration of cytokines and ICM-proteins combined with clinical characteristics for the sub-cohort of 60 subjects where cytokine and ICM-levels were analyzed. The models were also run on the full cohort of 128 subjects, using concentration of allergen-specific IgE combined with clinical characteristics as the predictor variables. In accordance with the univariable analyses, none of the multivariable models were able to confidently distinguish a clear pattern for classification of subjects into improved or non-improved (Fig. 4). When supplying the model with all variables available in the sub-cohort the best performing model was LGBM with an AUC of 0.59. The best results with an AUC of 0.63 were seen using a neural network using clinical characteristics and concentration of allergen specific IgE as variables in the full cohort.

### Cluster analysis

Finally, four different algorithms were used to cluster the subjects, solely based on protein expression (both Olink- and ICM-proteins) without regard to the AIT-results. All algorithms divided the subjects into three clusters. When comparing the results from the different algorithms, 48/60 subjects (80%) were consistently clustered together and thus were deemed to belong to 1 of 3 consensus clusters. The remaining 12 subjects were categorized as “no cluster”.

**Fig. 5:**
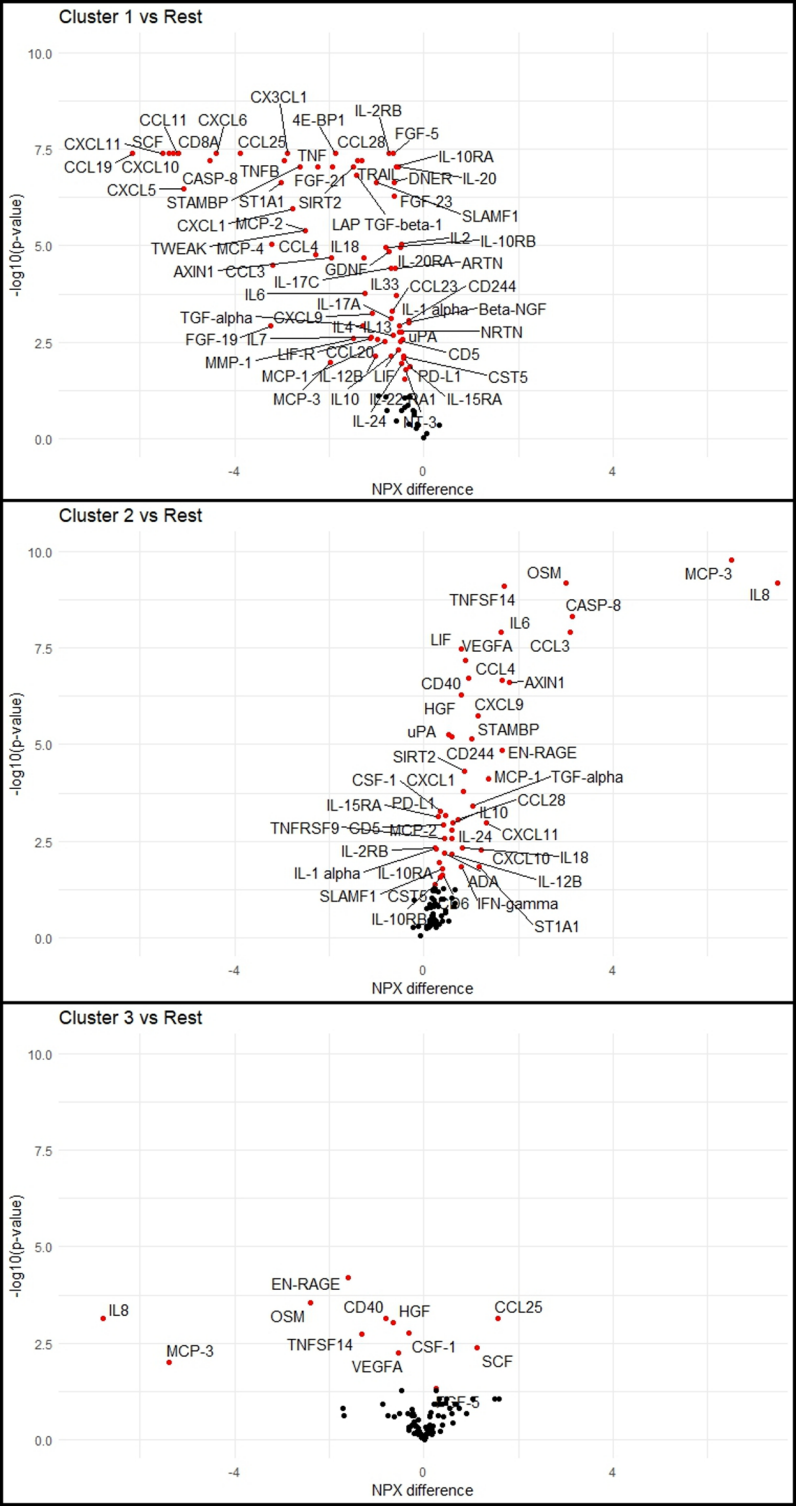
Differential expression of Olink proteins between consensus clusters. Differences presented as NPX difference, where 1 NPX difference is equal to a twofold change. *P* values from Wilcoxon rank sum test, adjusted with FDR. Colors represent statistical significance (red: *P* value < 0.05).

**Fig. 6:**
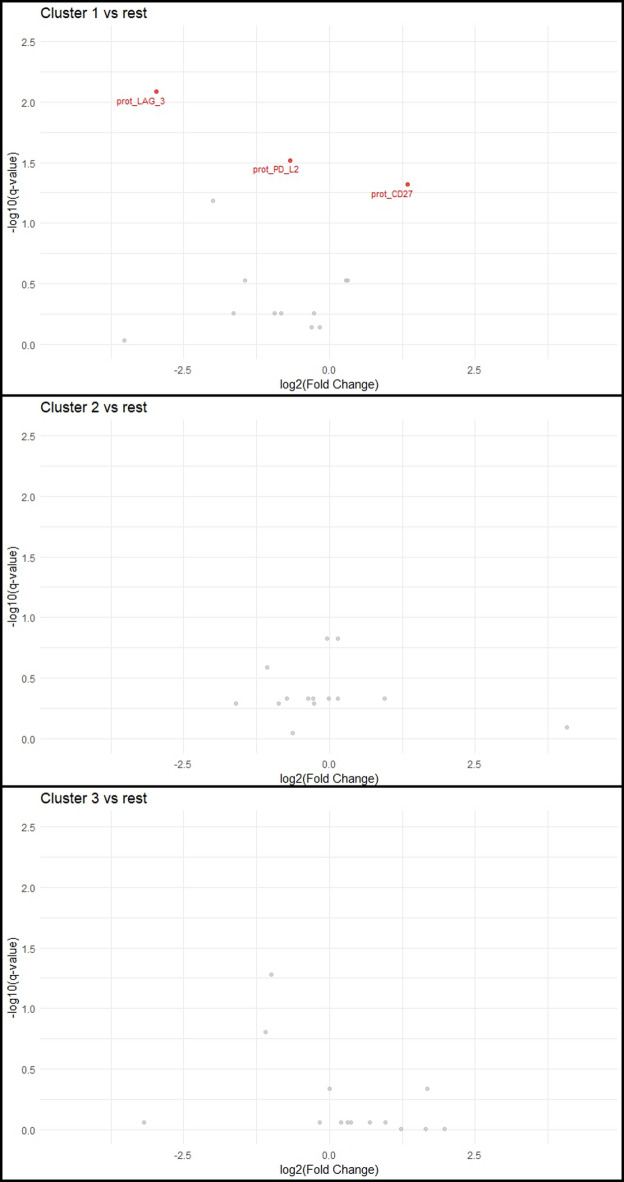
Differential expression of immune checkpoint molecule proteins between consensus clusters. Differences presented as log2(fold change). *P* values from Wilcoxon rank sum test, adjusted with FDR. Colors represent statistical significance (red: *P* value < 0.05).

No differences in clinical characteristics could be seen between the 3 consensus clusters (Supplement 5). To compare protein expression between the 3 clusters, univariable analysis of protein levels was performed between the clusters (Figs. 5 and 6). Between cluster 1 (n = 8) and clusters 2 + 3 (n = 40), a total of 72 Olink-proteins and 3 ICM’s were significantly different (fold change > 1.2; q-value < 0.05). Between cluster 2 (n = 15) and clusters 1 + 3 (n = 33), 45 Olink proteins were differentially expressed and between cluster 3 (n = 25) and clusters 1 + 2 (n = 23) 12 Olink proteins were significantly different.

## Discussion

Our aim for this study was to evaluate serum concentration of cytokines and ICM as predictors for AIT-success. Neither univariable nor multivariable analysis could find any correlation between AIT-response and protein levels in our study population.

It is well known that AR is a heterogeneous condition, both in terms of clinical characteristics and in how patients respond to different treatments^2,20^. One potential reason for this heterogeneity could be that there are many different immunological mechanisms and pathways that leads to the clinical presentation of what we call AR. As cytokines are important mediators of immune communication^21^, the measurement of cytokines and ICM creates a possibility to measure these differences in immunological mechanisms. For example, studies have previously shown differences in cytokine profiles between different severities of AR^22^.

One study have previously suggested that serum IL10 and IL33 concentrations might serve as a biomarker for AIT success, as they found IL10 and IL33 levels to be higher in non-responders compared to responders^16^. In our study, the serum levels of IL10 and IL33 were not statistically significantly different between improved and non-improved. In fact, the concentrations were slightly higher in the improved group, which contrasts with the previously mentioned study. The reason for this could be differences between the studies, mainly that the earlier study examined subjects treated with sublingual AIT against HDM-allergy, while in the present study we examined subjects treated with subcutaneous AIT against grass and/or birch pollen. It is possible that differences in the immunological mechanisms between these types of allergies plays a role in the different findings. It is also possible that technical limitations of this study, such as sample handling or our relatively small sample size, have limited our power to detect modest effects. Furthermore, the mentioned study was conducted in China, while our study is done entirely based on patients living in Sweden, and therefore it is possible that the differences in results are due to environmental factors. To our knowledge, this is the first study to examine cytokines in relation to AIT-results in a Swedish population.

Some cytokines of extra interest are cytokines that novel biological treatments are targeting, which are mainly IL4, IL5 and IL13^12^. However, none of these cytokines showed any correlation to AIT-efficacy in this study. Beyond IL4, IL5 and IL13, none of the 92 cytokines or 14 ICMs demonstrated statistically significant differences between groups after correction for multiple testing. This indicates that no individual protein in this panel showed discriminatory ability.

In an ideal world, there would be a single biomarker to easily predict which patients would benefit from AIT. However, due to the complexity of allergic diseases, it is unlikely that any such single biomarker exists. Instead, it is more likely that a combination of several biomarkers, possibly supplemented with clinical characteristics of the patient, might be able to predict AIT-success. When dealing with many variables, prediction models using machine learning can allow us to find complex correlations that would be hard to find using, for example, conventional regression analysis. In this study, we used 6 different machine learning algorithms, to be able to compare the abilities of the different models in predicting AIT-success using our data. We also ran the models with different variable sets to compare the performance when using only cytokine levels, cytokine levels in conjunction with ICM-levels and clinical characteristics, or IgE levels and clinical characteristics as the predictor variables. The best performing model when analyzing the sub-cohort with available data for cytokine and ICM-concentrations, showed an AUC of 0.59, meaning that it was able to correctly classify 59% of the subjects which indicate near-random performance, consistent with insufficient signal in the biomarker data for predictive patterns. Notably, the highest AUC (0.63) was obtained from models using only clinical characteristics and IgE. This further demonstrates that cytokine and ICM data did not improve predictive performance. In addition, PCA also failed to show separation by clinical variables also suggesting that basal cytokine and ICM profiles do not reflect major clinical phenotypes within this cohort.

When using clustering algorithms to stratify our subjects into clusters with different protein expression, 80% of the subjects were consistently grouped together indicating that the similarities and differences within and between the consensus clusters are strong as they were identified by all four algorithms. It seems that in general the studied cytokines were under-expressed in cluster 1 and overexpressed in cluster 2 (Fig. 5). It is important to note however, that since we could not see any correlation between clinical characteristics between the subjects in the resulting clusters, it is impossible to tell if the clusters correspond to a clinical or biological aspect in the study population, as they could just as well correspond to any technical or methodological differences or even random variations between the subjects.

One possible explanation for the lack of differences in cytokines and ICM in our material could be that the concentrations of these molecules at one single point in time is not that interesting, but rather that it is the dynamic in how these proteins are expressed in response to an allergen that dictates how the individual responds to AIT. In this study, we only analyzed serum concentrations of cytokines and ICM in blood samples collected before initializing AIT. An alternative approach would be to measure changes in the concentrations during AIT, as this might be able to identify non-responders at an earlier stage and therefore stop treatment if there is only a small chance of success. While this would not help to select which patients to offer AIT, it would save resources compared to completing 3 years of AIT before being able to evaluate the effect.

One limitation of this study is the fact that the blood samples were collected from the subjects at different times. The recruitment for the original cohort was conducted at the ENT-department at Örebro University Hospital during 1999–2015, where patients who were visiting the clinic to be evaluated for AIT was asked to be included. Upon their approval, they would leave a blood sample in conjunction with the clinic visit. Therefore, the timing of when the blood samples were drawn varies not only through the years 1999–2015, but also in what season they were collected. It is possible that, for example, the pollen levels during the time that the sample was taken could influence the results from the analysis. If such an effect was present and strong enough, it might conceal a weaker correlation between protein concentrations and AIT-effect. This is something that we could not correct for, as we did not have information about what season the blood samples were drawn.

Another important factor to consider is how the storage of the blood samples might have affected the results. Compared to larger molecules, such as IgE-proteins, both cytokines and ICM are more sensitive molecules, and it can be expected that the measured concentrations could be influenced by the fact that the samples have been frozen before analysis. Therefore, instability of many cytokines at − 20 °C, particularly with long-term storage, may introduce degradation as a potential source of measurement error. Furthermore, the duration of storage differs widely between the subjects in the study, as samples were drawn upon inclusion in the study, which was ongoing between 1999 and 2015. One way to estimate the validity of the results would be to compare the results to earlier studies that have used the same methods. For Olink-proteins, this is made difficult as the results are received as relative protein concentrations, and concentrations can therefore not easily be compared between different project runs without using bridging samples^23^. For ICM, the concentration levels and ranges we found are around the same levels found in another study using the same method to analyze ICM^24^. However, the inter-individual variation in soluble ICM concentrations observed in the present study may reflect biological heterogeneity or technical factors such as long-term storage, which could reduce the ability to detect true associations. In a future study, one could consider to further match samples based on time-spent-in-freezer, to minimize this potential confounder.

Another possible confounder when analyzing cytokine- and ICM-levels are medications that could influence these protein levels. We found no correlation between cytokine or ICM-levels and pre-AIT treatment with oral antihistamines, intranasal corticosteroids or systemic steroids. In the present study, only a small number of the subjects had a confirmed asthma diagnosis, and therefore the available data for asthma medication was deemed too small to make any meaningful analysis of the correlation between asthma medication and cytokine or ICM-levels. As the number of confirmed asthma patients were similar between the outcome groups, we do not believe that this has influenced our results in a meaningful way. However, it is nevertheless a potential confounder that future studies would benefit to control for.

This study was limited in how many of the 128 subjects in the full cohort could be further analyzed for cytokines and ICM. This was in part due to practical issues, such as insufficient serum volume remaining after previous analyses, but also due to economic considerations, as the analysis of cytokines and soluble ICM are relatively expensive and not routinely available in clinical use. It is possible that our limited sample size reduced the possibility of discovering differences between the improved and non-improved subjects. This is partly shown in the machine learning classification, where the best performing models were the models that had access to the full cohort, despite being limited to fewer variables as concentration of Olink-proteins and ICM was not available for the full cohort.

Furthermore, the limitations of the original cohort obviously affect this study as well. These limitations have been described in an earlier publication by the authors^6^, but consists mainly of a low response rate to the study questionnaires on which the AIT-response are based, as well as variations in the time between AIT and follow-up.

## Conclusion

This study did not demonstrate any evidence of an association between AIT outcomes and pre-AIT concentrations of cytokines or soluble ICM. Consequently, our results do not support the hypothesis that these biomarkers can reliably predict the clinical efficacy of AIT. The absence of association indicates that the immunological mechanisms underlying AIT response are likely more complex and influenced by multiple factors. Future research should include larger and more heterogeneous study populations, as well as longitudinal sampling. By analyzing samples collected at multiple time points, temporal changes in the immune response can be elucidated, providing insights into treatment effects and disease progression, which may ultimately improve our ability to identify predictors of treatment success and personalize AIT strategies.

## Supplementary Information

Below is the link to the electronic supplementary material.

Supplementary Material 1

## Data Availability

Due to sensitivity of health data, the dataset used in this study is not publicly available. It is, however, available from the corresponding author at reasonable request.
