# Supplementary material for: Cytokines and immunologic checkpoint molecules in predicting success of allergen immunotherapy
Source: Sci Rep. 2026 May 18;16:15356. doi: 10.1038/s41598-026-53894-6 (PMC13184136; doi:10.1038/s41598-026-53894-6)
Supplement: Supplementary file 1 — Supplementary Material 1 [file 41598_2026_53894_MOESM1_ESM.pdf]

Supplement 1 – list of cytokines included in Olink-analysis

Adenosine deaminase (**ADA**)

Artemin (**ARTN**)

Axin-1 (**AXIN1**)

Beta-nerve growth factor (**NGF**)

C-C motif chemokine 13 (**CCL13**)

C-C motif chemokine 19 (**CCL19**)

C-C motif chemokine 2 (**CCL2**)

C-C motif chemokine 20 (**CCL20**)

C-C motif chemokine 23 (**CCL23**)

C-C motif chemokine 25 (**CCL25**)

C-C motif chemokine 28 (**CCL28**)

C-C motif chemokine 3 (**CCL3**)

C-C motif chemokine 4 (**CCL4**)

C-C motif chemokine 7 (**CCL7**)

C-C motif chemokine 8 (**CCL8**)

C-X-C motif chemokine 10 (**CXCL10**)

C-X-C motif chemokine 11 (**CXCL11**)

C-X-C motif chemokine 5 (**CXCL5**)

C-X-C motif chemokine 6 (**CXCL6**)

C-X-C motif chemokine 9 (**CXCL9**)

CUB domain-containing protein 1 (**CDCP1**)

Caspase-8 (**CASP8**)

Cystatin-D (**CST5**)

Delta and Notch-like epidermal growth factor-related receptor (**DNER**)

Eotaxin (**CCL11**)

Eukaryotic translation initiation factor 4E-binding protein 1 (**EIF4EBP1**)

Fibroblast growth factor 19 (**FGF19**)

Fibroblast growth factor 21 (**FGF21**)

Fibroblast growth factor 23 (**FGF23**)

Fibroblast growth factor 5 (**FGF5**)

Fms-related tyrosine kinase 3 ligand (**FLT3LG**)

Fractalkine (**CX3CL1**)

Glial cell line-derived neurotrophic factor (**GDNF**)

Growth-regulated alpha protein (**CXCL1**)

Hepatocyte growth factor (**HGF**)

Interferon gamma (**IFNG**)

Interleukin-1 alpha (**IL1A**)

Interleukin-10 (**IL10**)

Interleukin-10 receptor subunit alpha (**IL10RA**)

Interleukin-10 receptor subunit beta (**IL10RB**)

Interleukin-12 subunit beta (**IL12B**)

Interleukin-13 (**IL13**)

Interleukin-15 receptor subunit alpha (**IL15RA**)

Interleukin-17A (**IL17A**)

Interleukin-17C (**IL17C)**

Interleukin-18 (**IL18**)

Interleukin-18 receptor 1 (**IL18R1**)

Interleukin-2 (**IL2**)

Interleukin-2 receptor subunit beta (**IL2RB**)

Interleukin-20 (**IL20**)

Interleukin-20 receptor subunit alpha (**IL20RA**)

Interleukin-22 receptor subunit alpha-1 (**IL22RA1**)

Interleukin-24 (**IL24**)

Interleukin-33 (**IL33**)

Interleukin-4 (**IL4**)

Interleukin-5 (**IL5**)

Interleukin-6 (**IL6**)

Interleukin-7 (**IL7**)

Interleukin-8 (**CXCL8**)

Interstitial collagenase (**MMP1**)

Kit ligand (**KITLG**)

Leukemia inhibitory factor (**LIF**)

Leukemia inhibitory factor receptor (**LIFR**)

Lymphotoxin-alpha (**LTA**)

Macrophage colony-stimulating factor 1 (**CSF1**)

NAD-dependent protein deacetylase sirtuin-2 (**SIRT2**)

Natural killer cell receptor 2B4 (**CD244**)

Neurotrophin-3 (**NTF3**)

Neurturin (**NRTN**)

Oncostatin-M (**OSM**)

Programmed cell death 1 ligand 1 (**CD274**)

Protein S100-A12 (**S100A12**)

Protransforming growth factor alpha (**TGFA**)

STAM-binding protein (**STAMBP**)

Signaling lymphocytic activation molecule (**SLAMF1**)

Stromelysin-2 (**MMP10**)

Sulfotransferase 1A1 (**SULT1A1**)

T-cell differentiation antigen CD6 (**CD6**)

T-cell surface glycoprotein CD5 (**CD5**)

T-cell surface glycoprotein CD8 alpha chain (**CD8A**)

Thymic stromal lymphopoietin (**TSLP**)

Transforming growth factor beta-1 proprotein (**TGFB1**)

Tumor necrosis factor (**TNF**)

Tumor necrosis factor ligand superfamily member 10 (**TNFSF10**)

Tumor necrosis factor ligand superfamily member 11 (**TNFSF11**)

Tumor necrosis factor ligand superfamily member 12 (**TNFSF12**)

Tumor necrosis factor ligand superfamily member 14 (**TNFSF14**)

Tumor necrosis factor receptor superfamily member 11B (**TNFRSF11B**)

Tumor necrosis factor receptor superfamily member 5 (**CD40**)

Tumor necrosis factor receptor superfamily member 9 (**TNFRSF9**)

Urokinase-type plasminogen activator (**PLAU**)

Vascular endothelial growth factor A (**VEGFA**)

Supplement 2 – list of proteins included in immunologic checkpoint molecule-analysis

B- and T-lymphocyte attenuator (**BTLA**)
Cluster of differentiation 27 (**CD27**)
Cluster of differentiation 28 (**CD28**)
Cluster of differentiation 80 (**CD80**)
Cluster of differentiation 137 (**CD137**)
Cluster of differentiation 152 (**CD152/CTLA**)
Glucocorticoid induced TNFR-related protein (**GITR**)
Herpesvirus entry mediator (**HVEM**)
Indoleamine 2,3-dioxygenase (**IDO**)
Lymphocyte activation gene 3 (**LAG-3**)
Programmed cell death protein 1 (**PD-1**)
Programmed cell death 1 ligand 1 (**PD-L1**)
Programmed cell death 1 ligand 2 (**PD-L2**)
T-cell immunoglobulin and mucin-domain containing-3 (**TIM-3**)

Supplement 3

Immunologic Checkpoint Molecule	Improved	
	No	Yes
BTLA	802 (461, 1,185)	743 (641, 1,071)
CD27	3,624 (2,594, 4,531)	4,254 (2,654, 5,981)
CD28	182 (116, 459)	191 (154, 309)
CD80	401 (289, 633)	472 (353, 622)
CD137	86 (52, 153)	94 (53, 124)
CD152	26 (14, 36)	26 (20, 37)
Unknown	0	1
GITR	65 (37, 158)	67 (36, 123)
HVEM	13 (10, 22)	14 (10, 20)
IDO	30 (21, 67)	28 (21, 47)
LAG_3	47 (23, 124)	50 (24, 57)
PD_1	76 (53, 98)	72 (48, 134)
PD_L1	0.80 (0.47, 2.74)	0.88 (0.61, 1.37)
Unknown	12	13
PD_L2	5,374 (4,360, 6,210)	5,340 (4,226, 6,754)
TIM_3	1,436 (1,167, 1,809)	1,516 (1,313, 1,868)

Results from ICM-analysis. Values presented as median (1st quartile, 3rd quartile) in pg/ml. Results listed as "Unknown" are results where the values were below the lower limit of detection.

Supplement 4

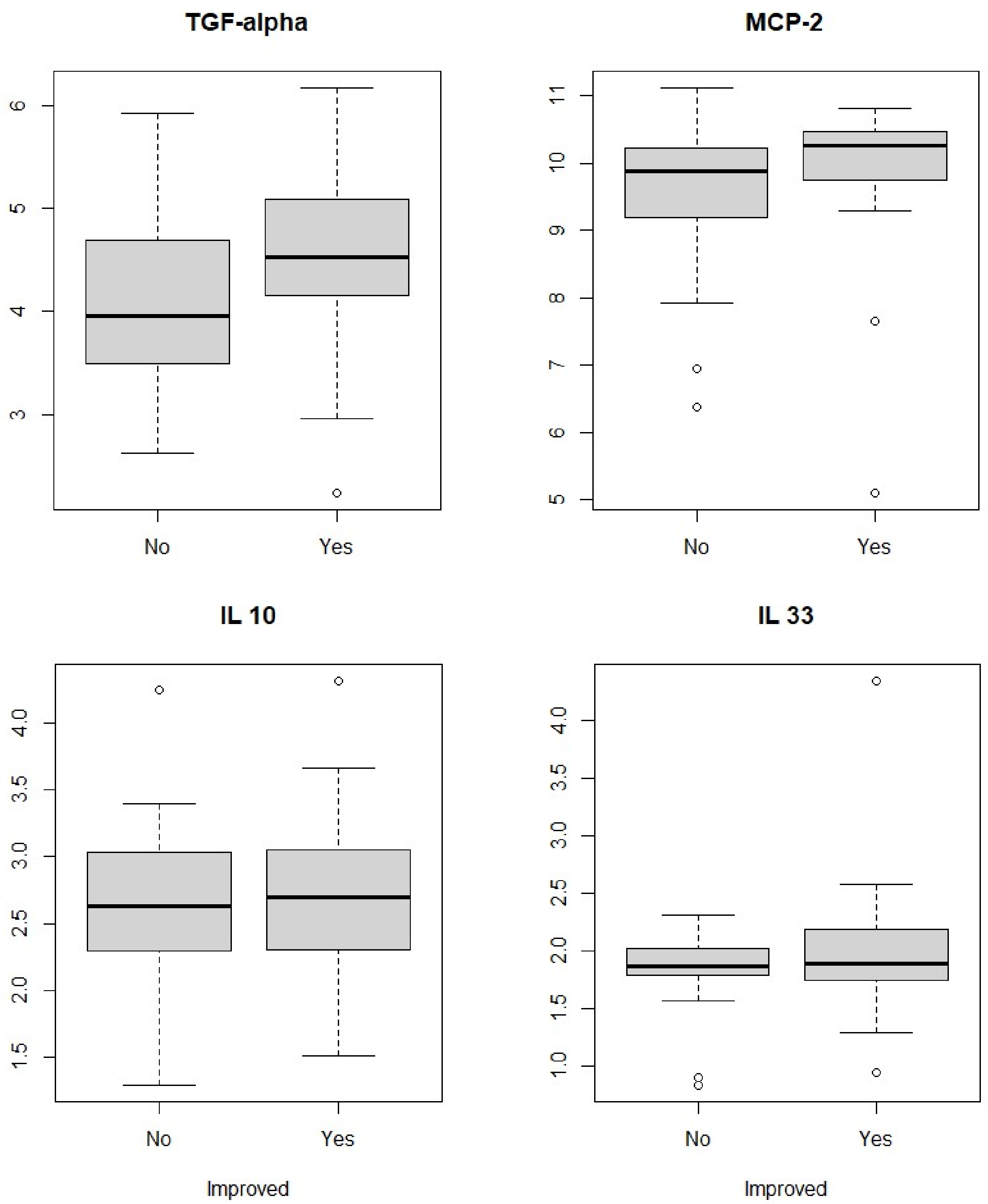

Boxplot of TGF-alpha, MCP-2, IL 10, and IL 13 compared between improved and non-improved subjects. Serum concentrations presented in Normalized protein expression (NPX). No statistically significant differences were seen between improved and non-improved subjects.

Supplement 5

Characteristic	Cluster1, N = 8[1]	Cluster2, N = 15[1]	Cluster3, N = 25[1]	NoCluster, N = 12[1]	p-value[2]
Improved	3 (38%)	9 (60%)	12 (48%)	6 (50%)	0.8
Sex					0.4
F	6 (75%)	8 (53%)	18 (72%)	6 (50%)	
M	2 (25%)	7 (47%)	7 (28%)	6 (50%)	
Age	31 (24, 39)	30 (19, 36)	24 (20, 37)	39 (26, 46)	0.2
Target allergen					0.2
B	3 (38%)	0 (0%)	6 (24%)	2 (17%)	
G	2 (25%)	6 (40%)	5 (20%)	5 (42%)	
GB	3 (38%)	9 (60%)	14 (56%)	5 (42%)	
Asthma	2 (25%)	2 (13%)	7 (28%)	5 (42%)	0.5
Self-reported Asthma	3 (38%)	5 (33%)	13 (52%)	7 (58%)	0.5
OAH	7 (88%)	14 (93%)	21 (84%)	10 (83%)	0.9
INCS	7 (88%)	12 (80%)	16 (64%)	10 (83%)	0.5
SCS	4 (50%)	6 (40%)	7 (28%)	2 (17%)	0.4
Skin pricktest					0.4
B	2 (29%)	0 (0%)	3 (12%)	1 (8.3%)	
G	0 (0%)	2 (13%)	4 (16%)	3 (25%)	
GB	5 (71%)	13 (87%)	18 (72%)	8 (67%)	
Unknown	1	0	0	0	

[1] n (%); Median (IQR)

[2] Fisher's exact test; Kruskal-Wallis rank sum test
